# The P2X1 receptor as a therapeutic target

**DOI:** 10.1007/s11302-022-09880-4

**Published:** 2022-07-11

**Authors:** Felix M. Bennetts, Jesse I. Mobbs, Sabatino Ventura, David M. Thal

**Affiliations:** Drug Discovery Biology, Monash Institute of Pharmaceutical Sciences, Monash University, Parkville, VIC Australia

**Keywords:** Structural biology, Drug discovery, ATP, Male contraception, Thrombosis and inflammation, Bladder dysfunction

## Abstract

Within the family of purinergic receptors, the P2X1 receptor is a ligand-gated ion channel that plays a role in urogenital, immune and cardiovascular function. Specifically, the P2X1 receptor has been implicated in controlling smooth muscle contractions of the vas deferens and therefore has emerged as an exciting drug target for male contraception. In addition, the P2X1 receptor contributes to smooth muscle contractions of the bladder and is a target to treat bladder dysfunction. Finally, platelets and neutrophils have populations of P2X1 receptors that could be targeted for thrombosis and inflammatory conditions. Drugs that specifically target the P2X1 receptor have been challenging to develop, and only recently have small molecule antagonists of the P2X1 receptor been available. However, these ligands need further biological validation for appropriate selectivity and drug-like properties before they will be suitable for use in preclinical models of disease. Although the atomic structure of the P2X1 receptor has yet to be determined, the recent discovery of several other P2X receptor structures and improvements in the field of structural biology suggests that this is now a distinct possibility. Such efforts may significantly improve drug discovery efforts at the P2X1 receptor.

## P2X1 receptors

### P2 receptors

P2 receptors have been identified in all human organ systems; they mediate a wide array of physiological responses [1, 2]. In humans, there are seven subtypes of the P2X ligand-gated ion channel (P2X1-7) and eight subtypes of the P2Y G protein-coupled receptor (P2Y1, P2Y2, P2Y4, P2Y6, P2Y11, P2Y12, P2Y13, P2Y14). Studies utilising selective antagonists and genetic knockouts have identified P2 receptors as potential targets for multiple conditions [3, 4]. However, only a small proportion of P2X and P2Y receptors are current targets of clinically successful medicines (Table 1). There is, thus, a clear need for more selective drug-like compounds to validate and progress clinical opportunities. This is particularly true for the subject of this review, the P2X1 receptor. Although the P2X1 receptor has been discussed in several exemplar reviews [4–9], it has often been overlooked in favour of other P2X subtypes. Herein, we review what is known about the P2X1 receptor as a drug target and the currently available ligands that target the receptor. We also highlight the potential impact that advances in structural biology could have on P2X1 receptor drug development.

**Table 1:** P2X and P2Y receptor ligands approved as drugs and their indications

Name	Class	Indication
Gefapixant	P2X3 receptor antagonist	Chronic cough
Clopidogrel, ticlopidine, ticagrelor, prasugrel and cangrelor	P2Y12 receptor antagonist	Thrombosis and stroke
Diquafosol	P2Y2 receptor agonist	Dry eye

### P2X receptors

**Fig. 1:**
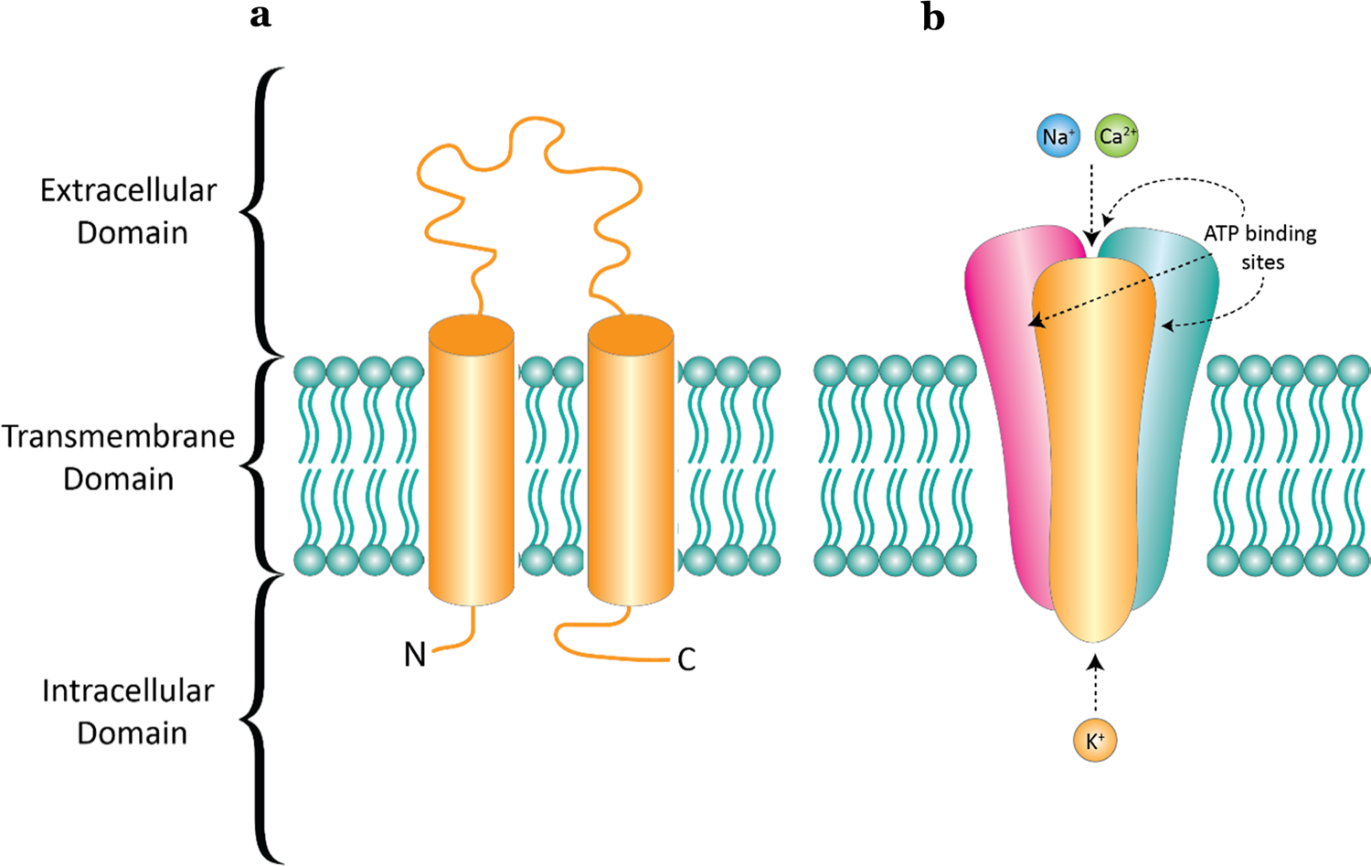
**a** A monomeric subunit of the P2X receptor in the plasma membrane and **b** a functional trimeric P2X receptor shown as a cartoon. There are three ATP binding sites located in the extracellular domain between subunits. Upon receptor activation by ATP binding, there is an inward flux of calcium and sodium ions and an outward flux of potassium ions

P2X receptors consist of three individual subunits that can be homotrimeric or heterotrimeric and together form a ligand-gated ion channel [10, 11]. Each P2X subunit contains two transmembrane spanning helices, a large extracellular domain and a small intracellular N- and C-terminus (Fig. 1a) except for the P2X7 receptor, which has a longer C-terminus. Endogenous ATP activates P2X receptors by binding to the three ATP binding sites that are located in the extracellular domain between subunits (Fig. 1b). Upon activation, P2X receptors form a non-selective pore that is permeable to cations; in the cellular context, these are typically calcium, potassium and sodium [12]. In addition, the P2X1, P2X2, P2X3, P2X4 and P2X7 receptors have been shown to transport large organic cations such as NMDG^+^ and spermidine across the membrane [8, 13–15]. The physiological importance of the transport of large cations has been difficult to study. However, a mutation in the P2X7 receptor which restricts large pore formation but not cation permeability was linked to chronic pain sensitivity [16]. The permeation of small cations, on the other hand, is critical in mediating specific cellular events. A few examples include smooth muscle contraction [17, 18], action potential propagation [19] and inflammation [20]. Following activation of P2X receptors, P2X1 and P2X3 receptors undergo rapid desensitisation while P2X2, P2X4, P2X5 and P2X7 receptors are minimally desensitised [12]. Trafficking studies show that the P2X1, P2X3, P2X4 and P2X7 receptors internalise upon activation and utilise the dynamin and clathrin-mediated pathways [21–25]. The P2X6 receptors are unique in that they have an uncharged N-terminal region that restricts the formation of functional homomeric channels and is retained within the endoplasmic reticulum [26]. P2X6 receptors can form functional heteromeric channels with P2X2, P2X4 and P2X7 receptors [11]. Glycosylation of P2X receptors is essential in the assembly and trafficking of functional receptors to the cell membrane [27–30], and enhanced P2X6 glycosylation has been shown to improve cell-surface expression and restore function [31]. In addition, the C-terminal of P2X receptors share a YXXXK motif that is important for membrane expression, while other regions, such as a tyrosine C-terminal motif at the P2X4 receptor and single residues within the C-terminus of the P2X7 receptor, control internalisation and trafficking [32–36]. Further information on the general properties of P2X receptors is available in a recent International Union of Basic and Clinical Pharmacology review [4].

### P2X1 receptors

The P2X1 receptor has a unique pharmacological profile that distinguishes it from other P2X receptors. For example, in heterologous systems, ATP exhibits the highest potency at the P2X1 receptor, with values ranging from 56 to 300 nM (Table 2). Additionally, only the P2X1 and P2X3 receptors are rapidly desensitised on a sub-second timescale [37]. This fast desensitisation has been shown to reduce the apparent potency of ATP for the P2X1 receptor [38]. Fluorescent labelling and cell surface biotinylation studies show that, in contrast to other P2X receptors, the P2X1 receptor is quickly internalised and recycles back to the membrane [25, 39, 40]. The P2X1 receptor also has higher fractional calcium currents than all other P2X receptors and is ranked highly among other ligand-gated ion channels for having high calcium permeability [41].

**Table 2:** Pharmacological properties of P2X1 receptor modulators. EC50 or IC50 values are recorded as nM values with respect to P2X1 activity. Letters in activity column designate P2X1 receptor origin with m for mouse, r for rat and h for human

Compound	P2X1 Activity (nM)	Source	Mode of action
ATP	56 (h), 100–300 (r)	[37, 73, 108]	Agonist
α,β-meATP	200 (h), 3200 (r)	[37, 108]	Agonist
2-MeSATP	54 (h), 100 (r)	[37, 108]	Agonist
BzATP	1.8 (h), 24,200 (r)	[37, 108]	Agonist
Ap6A	1120 (h), 600–720 (r)	[37, 73, 108]	Agonist
TNP-ATP	1 (r), 6 (h)	[108–110]	Antagonist
IP5I	3.1 (r)	[74, 108, 109]	Antagonist
Suramin	851 (h), 1700 (r)	[37, 108]	Antagonist
NF449	0.05 (h), 0.28–0.29 (r)	[75, 111, 112]	Antagonist
PPADS	1820 (h), 69 (r)	[37, 109]	Antagonist
PPNDS	14 (r)	[113]	Antagonist
MRS2159	9.4 (r)	[77, 109]	Antagonist
MRS2219	5900 (r)	[81]	PAM
Aurintricarboxylic acid (ATA)	8.6 (r)	[79]	Antagonist
PSB-2001	19 (h)	[78]	Antagonist
1	236 (m)	[76, 114, 115]	Antagonist
2	14,000 (r)	[80]	Antagonist
3	602 (h)	[116]	Antagonist
4	100,000 (h)	[117]	Antagonist
5	3000 (h)	[118]	Antagonist

There are functional roles for the P2X1 receptor in the urogenital, immune and cardiovascular systems. Within the urogenital system, P2X1 receptors localise to smooth muscle cells of various organs and tubules, including the vas deferens and bladder [1, 42]. Knockout studies in mice showed reduced fertility in male mice due to decreased vas deferens contractility, demonstrating the importance of the P2X1 receptor on the vas deferens [43, 44]. In the cardiovascular system, P2X1 receptors are primarily located on arterial smooth muscle, and P2X1 receptor knockout mice showed a slight increase in blood pressure [1, 42, 44, 45]. P2X1 receptors are also localised to immune cells such as platelets, macrophages, neutrophils and mast cells [1, 42, 46, 47]. Further studies in knockout mice established a functional role as mice exhibited impaired thrombus formation [48].

Like most P2X receptors, the quaternary P2X1 receptor structure can be in a heterotrimeric or homotrimeric form. Biochemical methods, ligand sensitivity and desensitisation kinetics have shown differences between the homotrimeric and heterotrimeric forms of the P2X1 receptors [11]. The P2X1/2, P2X1/4 and P2X1/5 heterotrimeric receptors have been co-purified and functionally verified in heterologous systems [11]. In native systems, heterotrimeric P2X1 receptors were expressed in low quantities making it difficult to ascertain their functional implications [49–51]. The homotrimeric form of the P2X1 receptor is likely the prevalent form, particularly in systems like the vas deferens where only the P2X1 receptor is expressed [44].

## P2X1 receptors as therapeutic drug targets

### Male contraception

P2X1 receptors and α_1A_-adrenoceptors are co-localised on the smooth muscle of the vas deferens [43]. Following activation of these receptors, there are contractions of the vas deferens, which propel spermatozoa anterograde through the vas deferens to the ejaculatory duct, where they mix with glandular secretions and are expelled during ejaculation [52]. If these receptors are blocked, sperm cannot leave their storage site in the cauda epididymis rendering a male infertile. Validation for this concept comes from a combined genetic knockout of the P2X1 receptor and α_1A_-adrenoceptor that produced complete infertility in a dual knockout male mice population [43]. Furthermore, the contraction of the human and mouse vas deferens is controlled by the same adrenergic and purinergic receptors suggesting that the contraceptive efficacy seen in mice could translate to humans [43, 53]. Equally important was the observation that the dual knockout male mice were sexually, physiologically and behaviourally healthy. There was concern that the P2X1 receptor and α_1A_-adrenoceptor knockout mice could have cardiovascular complications as both receptors are located on the smooth muscle of blood vessels and mediate vasoconstriction. Fortunately, the dual knockout mice had only minor changes in their baroreflex response, resting arterial pressure and heart rate similar to the changes seen in α_1A_-adrenoceptor knockout mice. In addition, clinical trials using α_1A_-adrenoceptor antagonists such as silodosin and tamsulosin as oral male contraceptives have shown promise [54–56]. This is encouraging, as α_1A_-adrenoceptor antagonists have been used chronically by men to treat benign prostatic hyperplasia and have proven to be safe and effective [57, 58]. However, pharmacological in vivo studies are needed to further validate the P2X1 receptor as a male contraceptive target. Nevertheless, the P2X1 receptor is a promising target for the design of a non-hormonal oral contraceptive.

### Thrombosis and inflammation

P2X1 receptors are also located on platelets and neutrophils, and within these cells, P2X1 receptors have important functional roles. P2X1 receptor activation can mediate platelet shape change, amplify platelet signalling and cause shear-induced aggregation of platelets [59, 60]. In mice models, pharmacologically blocking or genetically deleting the P2X1 receptor causes impairment in thrombus formation [48, 61]. Thrombosis is a cardiovascular condition in which a blood clot blocks blood flow. In theory, reducing platelet activation by antagonising the P2X1 receptor could alleviate thrombosis. P2X1 receptor activation also regulates activation and promotes chemotaxis of neutrophils [62, 63]. These two actions may be conflicting in inflammatory conditions, as seen in a model of acute colitis where P2X1 receptor knockout mice had high neutrophil levels, which contributed to thrombosis and intestinal bleeding [64]. In addition, studies into sepsis survival in pharmacological and genetic P2X1 receptor knockout mice have been conflicting, although most reported a reduction in survival rates signifying that the P2X1 receptor has a protective role [62, 65, 66]. Therefore, P2X1 receptors on neutrophils and platelets could be targeted for their regulatory role in severe inflammatory conditions such as sepsis. Further research is needed to describe if these conflicting functions of the P2X1 receptor will be detrimental for drug development. A functional role for the P2X1 receptor has also been identified for mast cells and macrophages, suggesting additional roles in inflammation and the immune system [46, 47].

### Bladder dysfunction

ATP and acetylcholine co-released from parasympathetic nerves stimulate smooth muscle contractions of the bladder from P2X1 and muscarinic receptors, respectively, which causes the voiding of urine [67]. Post junctional muscarinic receptors are typically targeted to treat bladder dysfunction, but purinergic contractions in bladder conditions such as overactive bladder and interstitial cystitis can be enhanced [68, 69]. Prolonged exposure of isolated detrusor muscle to α,β-meATP completely abolished purinergic contractions; hence, a P2X1 receptor antagonist could theoretically be used to abolish the purinergic component of the bladder contractions [70]. However, the P2X receptor antagonists suramin and PPADS were unable to completely inhibit the non-cholinergic contractions suggesting a role for another purinergic receptor [70]. As such, specific P2X1 receptor antagonists are needed to further validate the role of the P2X1 receptor as a target for bladder dysfunction.

## P2X1 drug discovery

### Medicinal chemistry

**Fig. 2:**
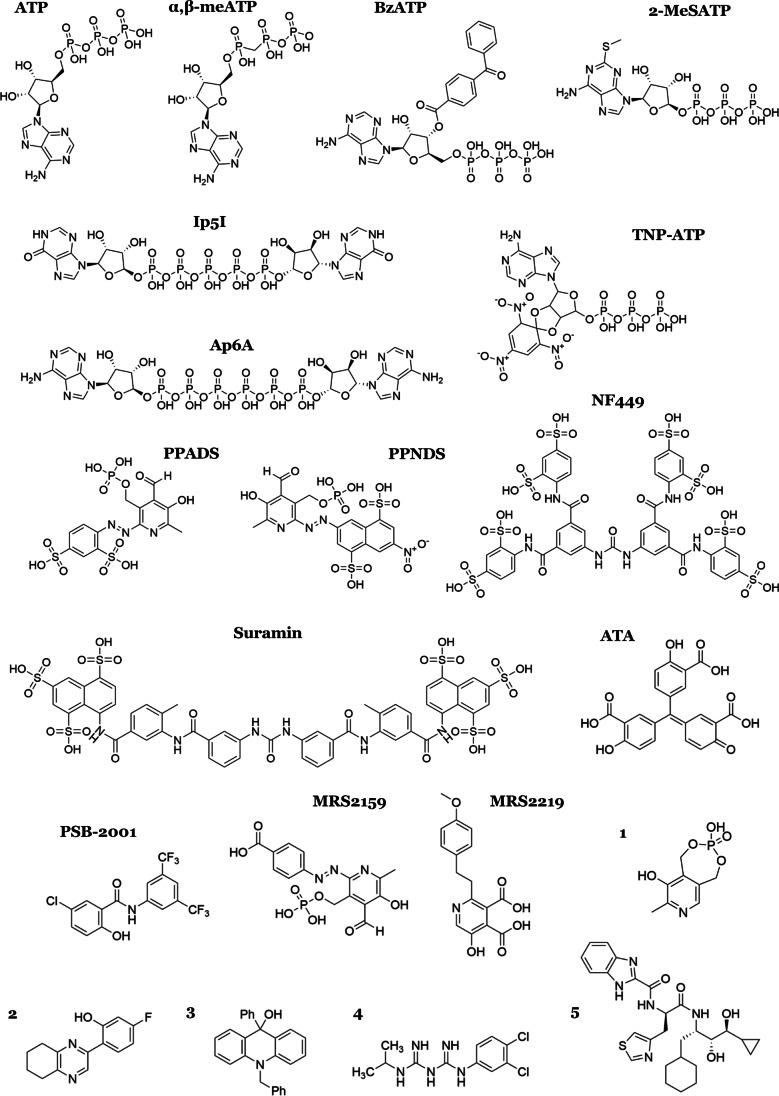
Chemical structures of P2X1 receptor modulators. P2X1 receptor modulators are referred to by their most common abbreviated name except for compounds 1–5 which do not have a common name and have been assigned a number

Many compounds have been shown to modulate P2X1 receptors with varying levels of selectivity and potency (Table 2, see for references). Derivatives of ATP have been designed to increase selectivity and reduce metabolic breakdown by ectonucleotidases. α,β-meATP is one such compound with high selectivity for the P2X1 and P2X3 receptor and has slower enzymatic breakdown than ATP [71, 72]. Another ATP derivative, BzATP, is the most potent agonist at the P2X1 receptor. Diadenosine polyphosphates and similar compounds can be both antagonists or agonists of the P2X1 receptor depending upon the length of the phosphate chain and the substituents on the adenosine group [73, 74]. The most potent P2X1 receptor antagonist is NF449 with sub-nanomolar potency at human and rat P2X1 receptors and is selective over other purinergic receptors [75]. Due to high molecular weight and polarity, these compounds are poor starting points for drug development (Fig. 2) [71]. Nevertheless, these compounds have been useful for in vitro and in vivo studies to investigate the function of the P2X1 receptor [17, 48]. PPADS is a selective P2 receptor antagonist whose drug properties were improved to create a series of antagonists, including MRS2159 and compound 1, which are low molecular weight compounds that are selective for the P2X1 receptor [76, 77]. Several research groups have identified novel P2X1 receptor antagonists. These antagonists have inhibitory activity in the range of low micromolar to low nanomolar potency and will hopefully become building blocks for molecules to be used in preclinical and clinical studies. Currently, MRS2159, aurintricarboxylic acid (ATA), PSB-2001 and compound 1 are the most exciting antagonists of the P2X1 receptor due to their nanomolar potency and low molecular weight. Furthermore, ATA, PSB-2001 and compound 2 are reported as non-competitive antagonists due to their pharmacological profile and molecular modelling studies [78–80]. Allosteric ligands are of great interest as it is likely easier to design selective ligands by not targeting the conserved ATP binding sites. In addition, a few studies have also looked into designing positive allosteric modulators (PAMs) that target the P2X1 receptor. So far, only MRS2219 has been identified as a small molecule PAM, although phosphoinositides and gintonin have been shown to potentiate P2X1 activation [5, 81, 82].

## P2X1 structural biology

### Structural biology of P2X receptors

Structural biology delivers a unique view into understanding the function of macromolecules on a molecular level. In particular, structural biology techniques are highly complementary to drug discovery efforts and can greatly facilitate the discovery and improvement of new therapeutics [83–85]. Prior to 2009, there were no available P2X receptor structures and mutagenesis studies were the primary method to study the key components of P2X receptors. However, since then, 29 P2X receptor structures have been deposited in the Protein Data Bank (PDB; Table 3). These structures were solved using nuclear magnetic resonance (NMR) spectroscopy (1 structure), X-ray crystallography (26 structures) and cryogenic electron microscopy (cryo-EM; 2 structures) with a resolution range between 2.7 and 4.1 Å. Recent advancements in 3D protein prediction have been significant, highlighted by AlphaFold predicting structures for the entire human proteome with reasonable accuracy for many proteins [86, 87].

**Table 3:** P2X receptor structures solved to date. Structures are characterised by their conformation, bound ligands, resolution, method of determination and length of the construct

Structure	Reference	Ligands’ bound and resolution	Receptor conformation	Method	Receptor construct
Zebrafish P2X4 receptor (zfP2X4)	[10, 92, 119]	**Apo** 2.9–3.5 Å **ATP** 2.8 Å **CTP** 2.8 Å	Closed, open	X-ray crystallography	Truncated
Rat P2X4 receptor (rP2X4)	[120]	N/A	N/A	NMR spectroscopy	Head domain
Gulf Coast tick P2X receptor (amP2X)	[121]	**ATP + Zn**^**2+**^ 2.9 Å	Pre-open	X-ray crystallography	Truncated
Human P2X3 receptor (hP2X3)	[89, 101, 94]	**Apo** 3.0 Å **ATP** 2.8–2.9 Å **2MeSATP** 3.1 Å **TNP-ATP** 3.3 Å **A317491** 3.1 Å **Gefapixant** 3.4 Å **ATP + Mg**^**2+**^ 3.8 Å **ATP + Ca**^**2**+^ 3.3 Å	Open, closed, desensitised	X-ray crystallography	Truncated
Panda P2X7 receptor (pdP2X7)	[102]	**Apo** 3.4 Å **ATP + A804598** 3.9 Å **A740003** 3.6 Å **GW791343** 3.3 Å **JNJ47965567** 3.2 Å **AZ10606120** 3.5 Å **A804598** 3.4 Å	Closed	X-ray crystallography	Truncated
Chicken P2X7 receptor (ckP2X7)	[122]	**TNP-ATP** 3.1 Å	Closed	X-ray crystallography	Truncated
Rat P2X7 receptor (rP2X7)	[88]	**Apo** 2.9 Å **ATP** 3.3 Å	Closed, open	Cryo-EM	Full length

**Fig. 3:**
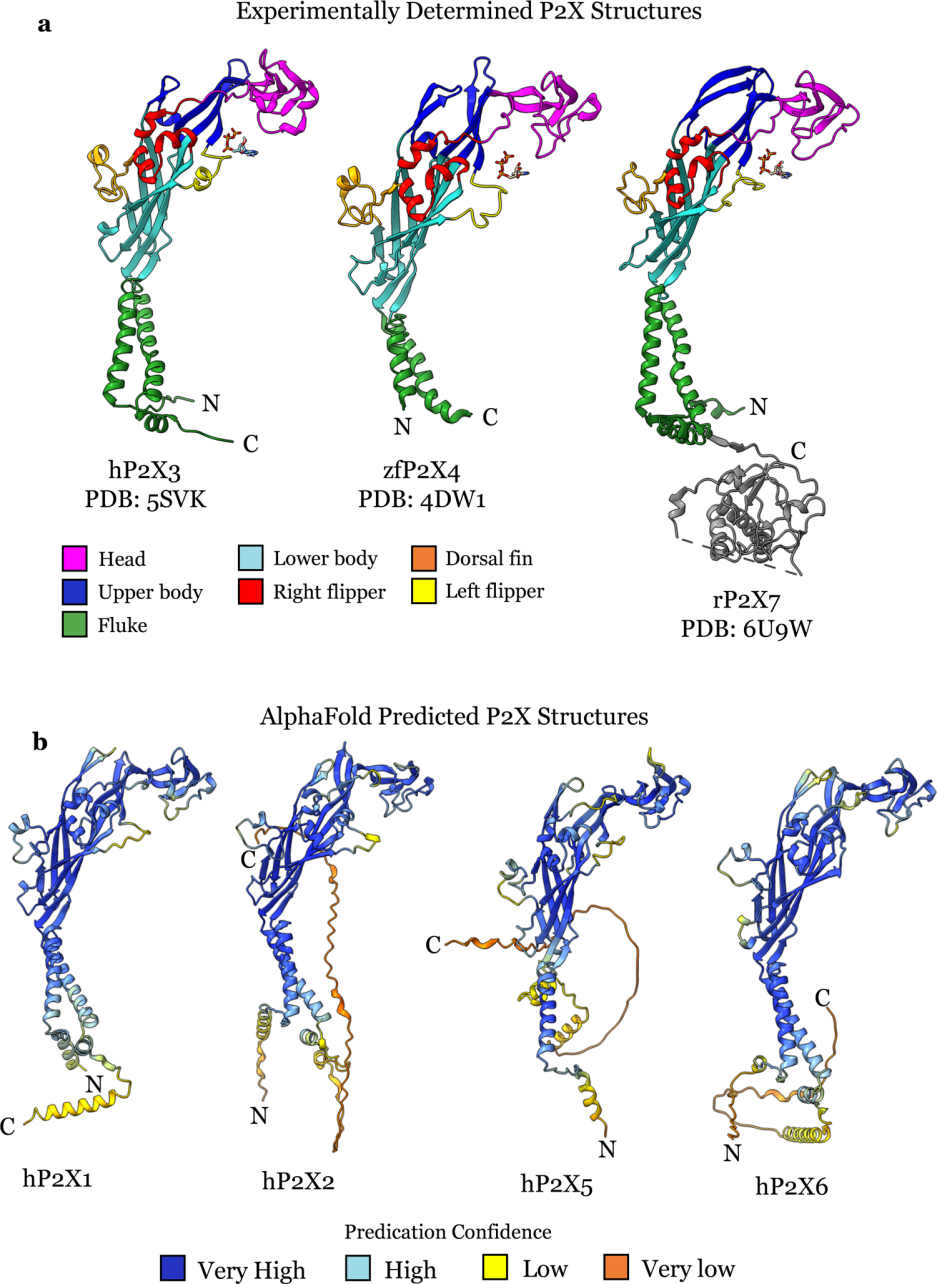
**a** Experimentally determined P2X structures coloured by features of a dolphin (based on the zfP2X4 [10]) with the head domain in pink, the upper body in dark blue, the lower body in light blue, the left flipper in yellow, the right flipper in red, the dorsal fin in orange and the fluke in green. **b** AlphaFold generated P2X receptor structures with each residue coloured by the confidence of the prediction with dark blue a very high confidence prediction, light blue a high confidence prediction, yellow a low confidence prediction and orange a very low confidence prediction

The primary structure of human P2X receptors (hP2X1-7) is between 388 and 595 amino acids and has 35–52% sequence similarity. The general structure of a P2X receptor was first described in the discovery of the zebrafish P2X4 receptor structure and the P2X4 subunit was zoomorphically described as the shape of a dolphin (Fig. 3a) [10]. Specifically, each P2X monomer can be described as having a head domain, left and right flipper, dorsal fin, upper body, lower body and fluke (Fig. 3a). All experimentally determined P2X receptor structures have exhibited the same structural architecture for the extracellular and transmembrane regions (Fig. 3a) [88, 89]. AlphaFold structure prediction of the P2X1, P2X2, P2X5 and P2X6 receptors also shows a similar architecture for the extracellular and transmembrane domains but not the intracellular region, this may reflect the fact that the N- and C-terminus are likely to be disordered, as all experimentally solved P2X receptor structures, except the cryo-EM P2X7 receptor structure, have truncated N- and C-termini (Fig. 3b) [86, 87].

### P2X1 receptor structure

Currently, there are no experimentally determined P2X1 receptor structures; however, reasonable assumptions can be made from other solved P2X receptor structures, molecular modelling, protein predication and mutagenesis of the P2X1 receptor. The P2X1 receptor AlphaFold model has a well predicted extracellular domain, orthosteric binding site and transmembrane domain (Fig. 3b). A recent study generated P2X1 receptor homology models using the experimentally determined zfP2X4, hP2X3 and pdP2X7 receptor structures [78]. This homology model compares well with the AlphaFold model of a single subunit alignment that has a root-mean-square deviation of 2.38 Å. P2X1 receptor models such as these could serve as valuable tools for modelling and structure-based drug design projects.

**Fig. 4:**
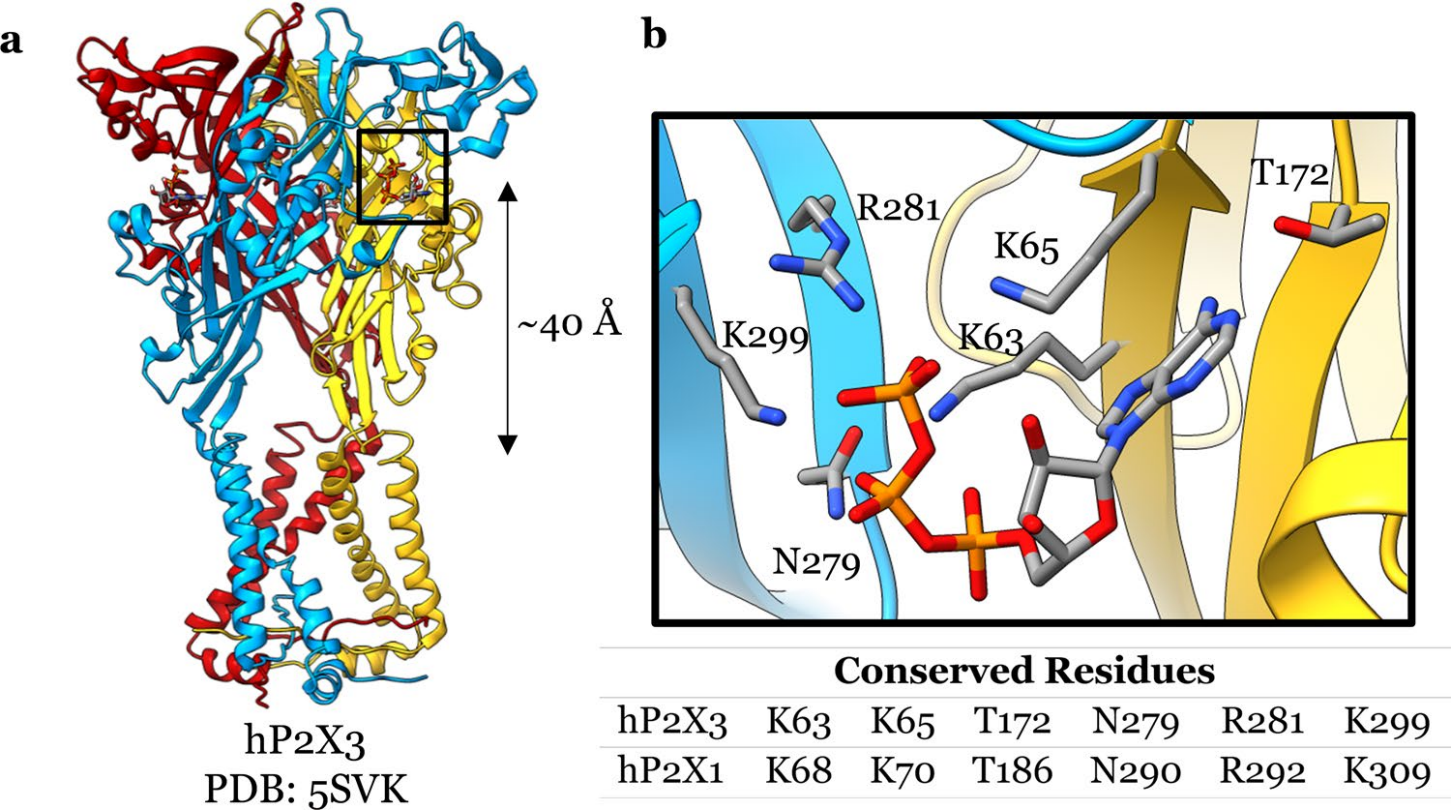
**a** ATP-bound hP2X3 receptor with one of the ATP binding locations emphasised (Protein Data Bank: 5SVK). **b** A close up view of the ATP molecule bound to the hP2X3 receptor with the interacting residues labelled, the interacting hP2X3 residues are reported in the table below with the sequence aligned P2X1 residues

Structures of P2X receptors have revealed three ATP binding sites where each monomer interfaces with the neighbouring monomer and is located approximately 40 Å above the transmembrane helices (Fig. 4a). The ATP molecules assume a U shape in the conserved orthosteric binding site (Fig. 4b). Mutagenesis of the ATP binding site in P2X1 receptors has revealed key residues. Alanine mutation of amino acid residues K68, K70, T186, N290, R292 and K309 in the P2X1 receptor caused large reductions in the potency of ATP [90, 91]. These residues are highly conserved, and ATP-bound P2X receptor structures reveal that these residues interact strongly with ATP (Fig. 4b) [88, 89, 92]. Several other P2X1 receptor residues have been linked to receptor activation, but most of these residues are unlikely to interact with ATP directly and instead may be important for either protein folding or the conformational rearrangement that occurs during activation (see review [93]). Another feature of the P2X1 orthosteric binding site may have been revealed by the hP2X3 receptor structures that contained a magnesium binding site adjacent to ATP [94]. This is supported by the ATP-Mg^2+^ complex being an agonist for the hP2X1 and hP2X3 receptors suggesting the magnesium binding site may also be located in the P2X1 receptor [95]. Although we have a good description of the ATP binding site for the P2X1 receptor, additional interactions could be uncovered by a high-resolution structure.

Channel activation shares a common mechanism among P2X receptor structures in which the dorsal fin and head domains move toward each other, producing a cleft closure, while the left flipper domain moves away from the orthosteric binding site. A P2X1 receptor mutagenesis study revealed that, unsurprisingly, the head domain is important in receptor activation. The movements of the dorsal fin and head domains produce an outward flexing of the rigid lower body and the beta-sheet strands of the lower body translate the movement to the transmembrane domains (TM). TM2 rotates counterclockwise, and there is an outward flexing that opens the pore and allows the permeation of cations. Mutagenesis studies of the P2X1, P2X2, P2X3 and P2X4 receptors have identified that polar and acidic residues on the outer edge of the transmembrane regions contribute significantly to calcium permeability; however, further studies are needed to explain what residues contribute to differing cation permeability among P2X receptors [41, 96–98]. Another region of interest for the P2X1 receptor is the cytoplasmic region, of which the N-terminus has been shown to control receptor desensitisation [99]. A study using cysteine mutagenesis and cross-linking compounds at the P2X1 receptor suggested that the cytoplasmic domain remains in a cap-like structure in both the apo and desensitised states [100]. The open state structure of the hP2X3 receptor revealed a cytoplasmic cap containing β-strands from the N- and C-terminus, which contain residues responsible for modulating receptor desensitisation [89]. Full-length P2X receptor structures would be useful in defining the differences among P2X receptors for receptor desensitisation and ion permeability.

P2X3, P2X4 and P2X7 receptor structures have revealed novel allosteric binding sites located in the extracellular domain of the P2X receptor [101, 102]. The hP2X3 receptor bound to gefapixant (other names: AF-219 and MK-7264) revealed an allosteric binding site located between the lower body and left flipper [101]. A series of structurally distinct ligands were crystallised in the pdP2X7 structure and revealed another allosteric binding site found in the upper body of the pdP2X7 receptor [102]. Using the zfP2X4 receptor structure to model in ligands complimented with mutagenesis studies, BX430 and 5-BDBD were demonstrated to bind the P2X4 receptor at an allosteric site located at the upper and lower body of the P2X4 receptor [103, 104]. There are other P2X receptor ligands that have been described as non-competitive but need to be further validated using mutagenesis and molecular modelling studies to verify their binding location. Unfortunately, most allosteric ligands for other P2X receptors have low potency for the P2X1 receptor, suggesting that these allosteric binding sites may not be completely conserved in the P2X1 receptor [105–107]. Fortunately, non-competitive antagonists for the P2X1 receptor have been recently discovered and molecular modelling studies have revealed two putative allosteric binding sites [78–80]. One allosteric site is located in the upper body of the P2X1 receptor using PSB-2001 which was docked into a homology model of the P2X1 receptor [78]. The other allosteric site is located between the lower body and left flipper as binding data demonstrated that ATA did not bind to the orthosteric binding site and molecular modelling showed that ATA docks at the same location as gefapixant in the hP2X3 receptor structure. Identifying and targeting allosteric binding sites is highly desirable for the P2X receptors as these are likely to represent better locations for designing specific ligands compared to the highly conserved orthosteric binding site in P2X receptors. Overall, many significant questions remain surrounding P2X1 receptor structure, function and drug discovery that are ready to be answered by experimental structures of the P2X1 receptor.

## Conclusion

The P2X1 receptor is still at the formative stage for the development of new therapeutics as promising (patho)physiology and clinical indications have been identified. The development of potent small-molecule ligands is progressing but has yet to produce the highly potent and well-validated compounds that are needed for in vivo studies. Improvement to the current suite of P2X1 receptor modulators is essential, which could be facilitated by structure-based discovery efforts. The recent determination of the P2X3, P2X4 and P2X7 receptor structures along with new models generated by AlphaFold provides a pathway toward the discovery of new P2X receptor structures [83, 84]. Ultimately, the P2X1 receptor needs better chemical tools to further validate its therapeutic potential in the treatment of thrombosis, inflammation, bladder dysfunction and as a male contraceptive.

## Data Availability

The data discussed can be found in the original publications referenced in the text.
